# Effect of Axis Change on Shrinkage Rate of 3D-Printed Bioceramic Zirconia Fabricated via Digital Light Processing

**DOI:** 10.3390/biomimetics10030140

**Published:** 2025-02-25

**Authors:** Ju-Young Park, Yoo-Na Jung, Kyoung-Jun Jang, Sang-Kyu Lee, Seong-Won Choi, Yong-Seok Lee, Yunzhi Peter Yang, Kwi-Dug Yun

**Affiliations:** 1 Department of Prosthodontics, School of Dentistry, Chonnam National University, Gwangju 61186, Republic of Korea; venus0439@naver.com (J.-Y.P.); yoonajung24@gmail.com (Y.-N.J.); 2 Research Institute, 3D Controls Co., Ltd., Busan 46721, Republic of Korea; jangkj@3dcontrols.co.kr (K.-J.J.); leesk@3dcontrols.co.kr (S.-K.L.); 3 Industry Support Center for Convergence Medical Devices, Chonnam National University Hospital, Gwangju 61469, Republic of Korea; magma1534@gmail.com; 4 Department of Mechanical Engineering, Myongji University, Yongin 17058, Republic of Korea; yslee23@mju.ac.kr; 5 Department of Orthopedic Surgery, School of Medicine, Stanford University, Stanford, CA 94305, USA; 6 Department of Materials Science and Engineering, School of Engineering, Stanford University, Stanford, CA 94305, USA; 7 Department of Bioengineering, School of Medicine, Stanford University, Stanford, CA 94305, USA

**Keywords:** 3D-printed zirconia, shrinkage rate, anisotropic, digital fixed prosthodontics

## Abstract

Isotropic shrinkage is critical for producing dimensionally accurate prostheses using zirconia. However, the anisotropic shrinkage of 3D-printed zirconia limits its utility in clinical applications. We aimed to evaluate the impact of specimen axis alterations on the shrinkage of digital light processing (DLP)-printed zirconia. Cubes measuring 10 × 10 × 10 mm^3^ (similar in size to molar crowns) and cuboids measuring 10 × 10 × 20 mm (similar in size to a three-unit bridge) were manufactured using a DLP 3D printer. Zirconia specimens were pre-sintered at 1300 °C and 1400 °C. The Z-axis of some specimens was switched to the X-axis before the final sintering procedure. The X-axis, Y-axis, and Z-axis lengths of the green body, pre-sintered block, and fully sintered block were measured using digital calipers. The 3D-printed specimens showed lower shrinkage and higher deviation than the milled specimens, whose shrinkage rate was 26%. The shrinkage rates of the 3D-printed cubic specimens were 19.9% (length), 20.0% (width), and 21.99% (height), while those of the cuboidal specimens were 20.26%, 19.72%, and 21.81%, respectively. For the 3D-printed specimens, which shrink anisotropically during sintering, the axis change step had no significant impact on the shrinkage rate. In all groups, the shrinkage rate along the building direction during printing significantly exceeded that along the gravity direction during sintering.

## 1. Introduction

Fixed dental prosthetics were originally fabricated with gold using a casting method. However, gold has now been completely replaced by zirconia. The fabrication of commercially available zirconia dental prostheses is a subtractive process performed using computer-aided design/manufacturing (CAD/CAM) systems. Zirconia, which is used to create the final prostheses in digital dentistry, shrinks during the sintering procedure. Controlling the rate of this shrinkage is essential for producing accurate prostheses. The conventional CAD/CAM-based production of zirconia prostheses begins with a fully sintered zirconia block, which enables the creation of relatively accurate prostheses; however, the long milling time and the reduced lifetime of the milling bur render this process economically disadvantageous. Currently, zirconia prostheses are produced using pre-sintered zirconia blocks, which are less hard and therefore require less milling time. However, the manufacturing of dental prostheses via milling involves several challenges, including high material consumption, excessive tool wear, a high possibility of microcracks due to surface defects in the manufactured prostheses, and difficulty in reproducing complex structures [1,2].

Additive manufacturing overcomes the aforementioned disadvantages, offering short manufacturing times, reduced manufacturing costs through material recycling [3], and simple processes. This method has therefore gained popularity in the manufacturing of not only metals and polymers, but also of complex ceramics [4].

Ceramic additive manufacturing is a high-precision technology that differs from conventional manufacturing methods used for ceramics [5,6]. The fabrication of ceramic components includes model design, slicing, printing, and post-treatment steps [7]. The printing step is further categorized into three types: direct inkjet-based printing, extrusion-based direct writing, and light exposure-based stereolithography [8]. Additionally, stereolithography is divided into laser scanning projection stereolithography (LSPS) and mask projection stereolithography (MPSL) methods [9]. LSPS involves drawing a laminate by emitting a laser beam at a single point, while MPSL involves liquid crystal display (LCD) light sources being emitted in a projector form and stacked layer by layer using digital light processing (DLP) technology [10]. Both methods are known to offer high accuracy and excellent surface finish [11,12]. However, DLP accelerates the printing process through a combination of ultra-fast switching and integral projection, in addition to providing a higher resolution [13] and the ability to cure an entire layer of material at once. Thus, DLP is advantageous for the fabrication of small components [14].

The 3D printing of zirconia is gaining interest in several fields due to its utility in the manufacturing of dental prosthetics, implants, scaffolds, and except these cutting blades and tools, as well as other mechanical applications. Whether in biological or mechanical applications, the final products must be manufactured with the correct dimensions. The 3D printing of zirconia via DLP involves the following steps: preparing the slurry, optimizing the printing parameters, and establishing appropriate debinding and sintering cycles. Among these, the debinding and sintering stages are critical to the dimensional accuracy of the final product. Dimensional changes occur during physical and chemical alterations, including the removal of porosity and grain enlargement. If the final product dimensions become predictable, 3D-printed zirconia can be extensively utilized [15,16].

The 3D printing of dental ceramics involves challenges in controlling shrinkage rate along with translucency, color properties, and accuracy [15,16]. Existing CAD/CAM methods for zirconia prostheses yield homogeneous particle distributions, resulting in uniform plastic shrinkage rates of approximately 25–30% on average. This is because pre-sintered zirconia particles are compressed uniformly in multiple directions before being cut and sintered [17]. Conversely, additively manufactured zirconia exhibits anisotropic compression during sintering, i.e., different levels of contraction along different axes, with distinct contraction patterns reported in different studies [18]. A cuboid with dimensions of 50 mm × 10 mm × 4 mm manufactured using a 40 vol% zirconia suspension showed shrinkage rates of 20–30% along each axis, with the shrinkage magnitude decreasing in the order of width, height, and length [19]. Li et al. measured the shrinkage rates of a 5 µm × 2.5 µm × 30 µm cuboid printed using a 45 vol% zirconia suspension to be 18.1%, 20%, and 24.3%, respectively [20]. Relative to the predicted theoretical isotropic shrinkage, additively manufactured ceramics tend to exhibit distortion during sintering [21], which can be attributed to various factors, including particle shape, structure, orientation, porosity, and gravitational effects [22,23,24,25,26]. We aimed to optimize the gravitational effects based on the growth principles of trees to induce isotropic shrinkage in zirconia. Trees regulate the distribution of their internal structure to minimize deformation, which allows them to grow uniformly upward despite gravitational influence. We intended to apply this principle to zirconia 3D printing by adjusting the sintering along the vertical axis to ensure uniform shrinkage.

Different shrinkage rates in different directions can compromise the dimensional precision of the final prosthesis. Studies have shown that 3D printing involves different shrinkage rates along different axes, with no method for isotropic shrinkage being available yet. Hence, we aimed to evaluate the effect of gravity on shrinkage during sintering and determine the feasibility of achieving isotropic shrinkage through axial adjustments during the sintering of a 49 vol% zirconia suspension through DLP. Our hypothesis was that changing the axes during sintering does not affect the shrinkage rate.

## 2. Materials and Methods

### 2.1. Experimental Groups

**Figure 1:**
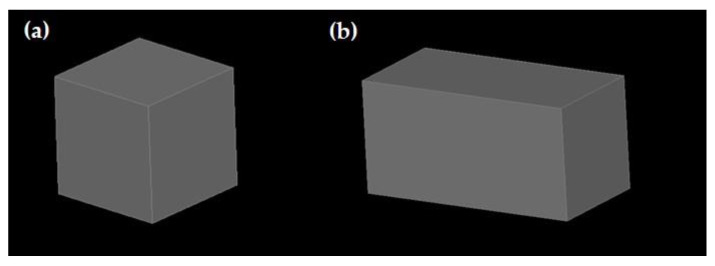
STL images of (**a**) cubic and (**b**) rectangular specimens.

The shrinkage rate was evaluated with respect to the manufacturing method, pre-sintering temperature, and the specimen axis during the sintering process. The specimen group manufactured via milling was labeled “M”. Among the 3D-printed groups, the cubic and cuboidal specimens were labeled “C” and “R”, respectively (Figure 1). The cubic and cuboidal specimens were designed to measure 10 mm × 10 mm × 10 mm and 20 mm × 10 mm × 10 mm (length × width × height), respectively, using Autodesk Fusion 360 (Autodesk, San Rafael, CA, USA), and the files were exported in a standard tessellation language (STL) format (Figure 1). The sizes of the cubic and cuboidal specimens are equivalent to those of a single molar and a three-unit molar bridge, respectively.

The milling group, serving as the control group, was manufactured using CAD/CAM methods currently employed in clinical practice. During the subtractive processing, the specimen (Arena, Arum Dentistry, Seoul, Republic of Korea) underwent 7.5% shrinkage from the green body to the preliminary sintering stage, and the manufacturer expected 18.73% shrinkage between the preliminary and final sintering stages. The specimen was fabricated from a pre-sintered block set to expand to 1.2312 times its original size, using a five-axis milling device (5X-450, Arum, Seoul, Republic of Korea).

Since the shrinkage rate can vary with the sintering temperature, its variation with respect to the specimen axis was evaluated for two pre-sinter temperatures: 1300 °C and 1400 °C. The corresponding groups were designated as “3” and “4”, respectively. The groups that underwent an axis change during sintering were additionally marked with an “X” (Table 1). Specifically, for these specimens, the Z-axis—which is greatly affected by gravity—was switched to the X-axis before the final sintering step.

### 2.2. DLP Conditions

The material used for printing was a 49 vol% zirconia suspension, in accordance with Jang et al. [27] (Table 2). The ceramic raw material used was zirconia powder (TZ-3Y, Tosoh, Tokyo, Japan), the binder in which was removed through a separate heat treatment. The zirconia particle phase was a monoclinic material containing 3 mol of a yttria stabilizer (Tosoh, Japan). The dispersant employed was BYK-180 (BYK-CHEMIE, Wesel, Germany). The relative density and porosity of the sintered zirconia specimen were 99.5% and 0.6%, respectively.

**Figure 2:**
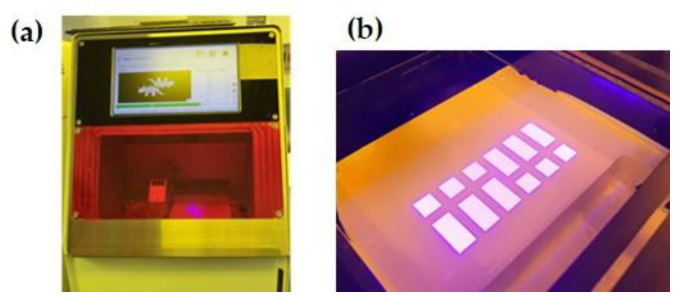
(**a**) DLP 3D printer. (**b**) Irradiation of ultraviolet light on zirconia suspension vat.

We employed the TD6 ceramic printer (3D Controls Co., Busan, Republic of Korea), a top-down DLP 3D printer that stacks layers of printing material on the platform as the platform descends one layer at a time. Radical photopolymerization was performed within a wavelength range of 385–400 nm, following the manufacturer’s instructions, and each layer was laminated to a thickness of 50 μm through light exposure for 10 s (Table 3 and Figure 2).

### 2.3. Axis Setting

**Figure 3:**
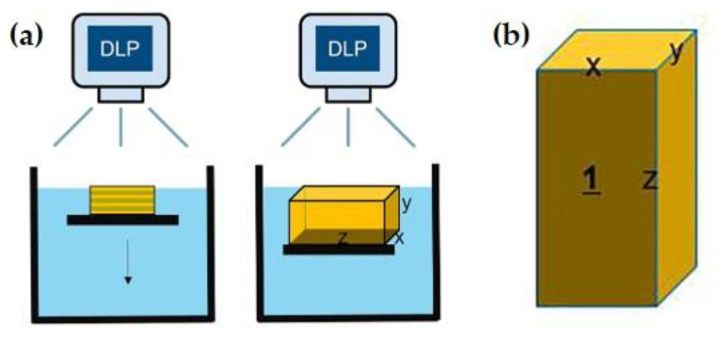
(**a**) Schematic of printing process. (**b**) Axes for shrinkage evaluation.

To specify the axes, the cubic and cuboidal specimens were positioned such that the bottom surface during 3D printing would be the frontal surface during sintering. The length, width, and height of the frontal surface were designated as the X-axis, Y-axis, and Z-axis, respectively (Figure 3).

**Figure 4:**
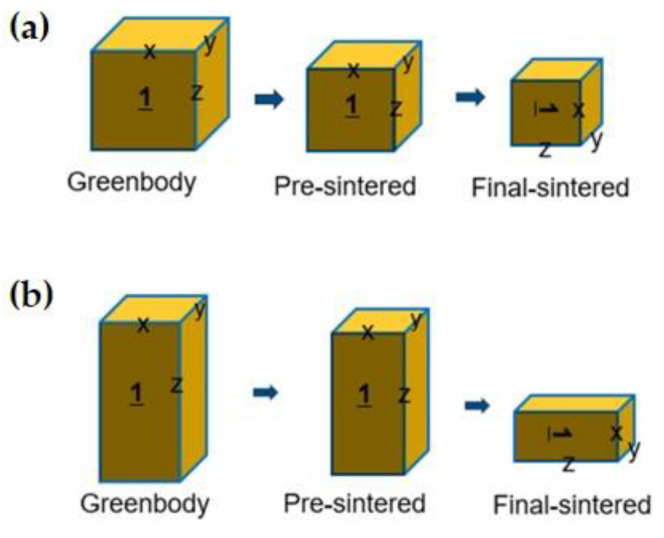
Schematic illustrating axis change during sintering: (**a**) cube; (**b**) cuboid.

For the groups that underwent an axis change during sintering, the axes were set after pre-sintering, with all specimens cooled to room temperature. Before the final sintering, the specimens were rotated to change the Z-axis into the X-axis (Figure 4).

### 2.4. Debinding and Sintering Schedule for 3D-Printed Specimens

**Figure 5:**
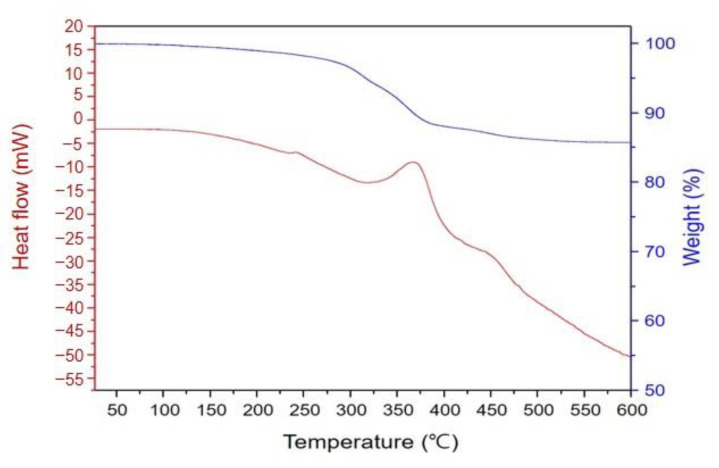
TGA showing mass change of specimens with increasing temperature. Weight decreased 14.89% as temperature increased to 600 °C. Red line represents heat flow, and blue line represents weight change.

According to thermogravimetric analysis (TGA), 230 °C, 380 °C, and 600 °C showed the weight loss and heat flow results. (Figure 5). Thus, the temperature was maintained at 230 °C, 380 °C, and 600 °C, where the heating reaction occurred in the differential scanning calorimeter during thermal weight analysis. The degassing schedule was optimized by checking for defects in the product.

The pre-sintering process was performed for 1 h after raising the temperature to 1040 °C at a heating rate of 1 °C/min. The specimens were then heated to 1300 °C or 1400 °C, depending on the experimental group, at a rate of 5 °C/min, before being cooled to room temperature. For the groups that underwent the axis change, the specimens were rotated 90° to right in this section and laid down before proceeding with the final sintering process.

During the final sintering process, the temperature was raised to 600 °C at 10 °C/min and maintained thereafter for 30 min. It was then raised to and maintained at 1040 °C for 1 h and then 1300 °C/1400 °C for 30 min. Subsequently, it was raised to 1500 °C at 5 °C/min and maintained at this value for 2 h (Table 4).

**Figure 6:**
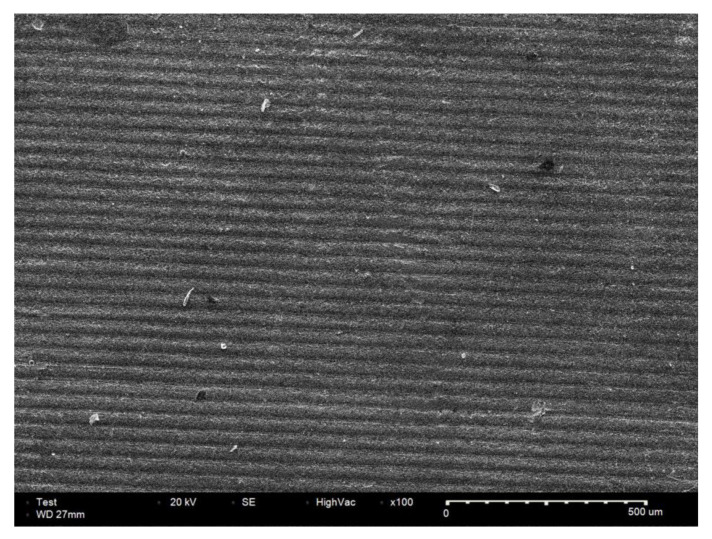
Scanning electron microscopy image of 3D-printed zirconia specimen after sintering.

Figure 6 shows the cross-section of a 3D-printed zirconia specimen after sintering. This specimen was printed in 25 μm thick layers, with no cracks or voids observed in the cross-section.

### 2.5. Shrinkage Rate Evaluation

The shrinkage rate of the zirconia specimens was assessed with respect to the manufacturing method. For the 3D-printed specimens, the shrinkage rate was evaluated based on the pre-sintering temperature and based on whether samples underwent an axis change after pre-sintering. The length of the sintered body was measured using a micrometer caliper accurate up to 0.001 mm. The measurements were recorded thrice, and the linear shrinkage rate was calculated using the following equation (ASTM C326):

∆L = (L_0_ − L)/L_0_ × 100,

where ∆L, L_0_, and L denote the linear sintering shrinkage (%), the pre-sintering specimen length, and the post-sintering specimen length, respectively.

### 2.6. Statistical Analysis

Statistical analysis was performed on the specimens using SPSS 23.0 (SPSS Inc., Chicago, IL, USA). Since each group comprised eight specimens, normality testing could not be performed to compare the shrinkage rates. Therefore, significance was tested using the nonparametric Kruskal–Wallis method, followed by a post hoc Dunn test. All results were evaluated at the adjusted significance level, with *p* < 0.05 considered statistically significant.

## 3. Results

### 3.1. Shrinkage Rate Evaluation Based on Manufacturing Method

**Figure 7:**
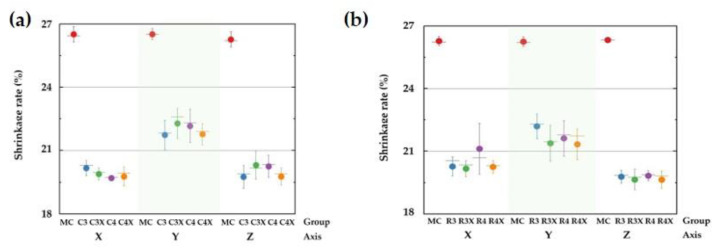
Mean and standard deviation of shrinkage rates for (**a**) cubic and (**b**) cuboidal specimens.

The subtractively processed zirconia specimens showed a 26% shrinkage rate along all axes. However, the 3D-printed cubic zirconia specimens showed 19.88%, 21.99%, and 20.02% shrinkage rates along the X-axis, Y-axis, and Z-axis, respectively. The average shrinkage rate for the milled group along all axes of the cubic and cuboidal specimens exceeded that for the 3D-printed group (Figure 7).

The cuboidal specimens displayed results similar to those of the cubic specimens. While the average shrinkage rate for the milled group exceeded that for the 3D-printed group, only the R3X and R4X specimens exhibited a significant difference in MR along all axes (Figure 7).

### 3.2. Shrinkage Rate Evaluation Based on Axis Change at Different Pre-Sinter Temperatures

**Figure 8:**
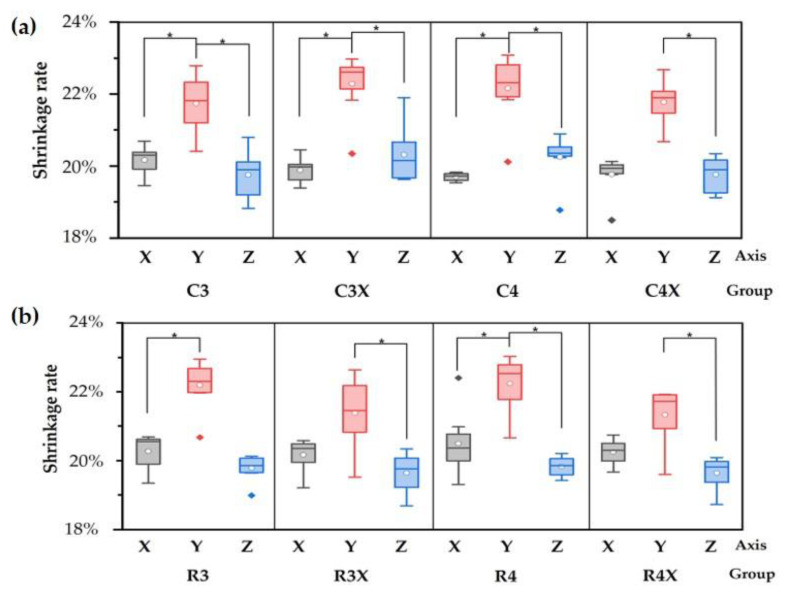
Shrinkage rate along each axis in each (**a**) cubic and (**b**) cuboidal specimen group.

No significant differences attributable to the pre-sintering temperature were observed for either the cubic or the cuboidal specimens. Similarly, the axis change process did not produce any significant difference. However, upon comparing the shrinkage rates by axis in the 3D-printed groups, anisotropic shrinkage was observed in all groups, with significantly higher shrinkage seen along the Y-axis (*p* < 0.05) than along the X-axis or Z-axis; moreover, the standard deviation was the highest for the Y-axis (* *p* < 0.001) (Figure 8).

## 4. Discussion

Subtractive processing, a method for manufacturing sintered ceramic products, is expensive, time-consuming, and unsuitable for complex-shaped parts [28]. By contrast, additive manufacturing is relatively inexpensive because it does not require milling tools, and it can also be used to create complex geometric structures, such as tooth cavities [29]. However, ceramics are currently not as widely used in additive manufacturing based on light-curing resin molding (LCM) as polymers or metals. This is because the drying, debinding, and sintering steps required for the ceramic material to achieve appropriate final characteristics and shape are highly challenging to integrate with additive manufacturing techniques [30]. According to German’s debinding theory [31], the debinding time and component size follow a right-angled square relationship. For instance, if the thickness of a part is doubled, the debinding time will be quadrupled. Therefore, the LCM method is desirable for the production of small and complex parts, such as dental prostheses.

The debinding of a zirconia component, which involves burning the resin binders, is a vital process that determines the final product quality. In this study, the zirconia green body underwent only an endothermic process without any loss of mass when the temperature was below 200 °C. However, between 200 and 300 °C, the mass began to decrease along with heat being radiated. Further, from 300 to 600 °C, 15% mass loss occurred along with the endothermic process. Accordingly, the debinding process can be divided into a low-temperature degassing section (200–300 °C) and a high-temperature degassing section (300–600 °C).

In the low-temperature debinding section, the resin binders reach their boiling point and partially melt; the gaseous products inside the zirconia green body are discharged to the outside, which reduces the heat flow; and the subsequent gaseous byproducts diffuse from the interior of the green body to the surface due to the heat and capillary force, thus forming interconnected pore channels within the green body. In this state, excessive heating rates can accelerate the diffusion of the gasses and cause cracks in the product. In the high-temperature debinding section, the binder decomposes rapidly, causing carbonization; the elemental carbon thus produced reacts with oxygen to generate carbon dioxide, which results in substantial heat releases. If the generated carbon dioxide and heat are not smoothly discharged from the interior to the exterior of the green body, cracks occur. At ~600 °C in our experiments, a large amount of heat was released again, although no change in mass was observed above this temperature; this indicated that the debinding process was completed [3].

When the temperatures corresponding to the mass loss peaks, identified through the TGA, were maintained, the molten resin binder and gaseous decomposition products could be smoothly discharged from the specimen, with stable interconnected pore channels being formed; this allowed the green body to quickly lose the binder without suffering cracks [32]. Based on the results, the optimized debinding process for 49 vol% zirconia involved maintaining the temperature at 230 °C for 1 h, at 380 °C for 2 h, and at 600 °C for 1 h, with a heating rate of 0.5 °C/min.

The space between the zirconia powder grains influences the shrinkage during sintering. The pore formation and grain connection in zirconia also affect the shrinkage rates along different directions [33]. During sintering, the zirconia crystals grow as the pores between the particles disappear and the particles bond, which causes the body to shrink. Thus, zirconia shrinkage is inevitable during the sintering process in additive manufacturing [34]. The shrinkage rate directly affects the dimensional accuracy of zirconia products, which is critical for the appropriate fitting of dental prostheses or implants.

The specimen subjected to subtractive processing in this study experienced a 7.5% shrinkage from the green body to the preliminary sintering stage, and the manufacturer expected an 18.7% shrinkage from the preliminary sintering to the final sintering stage. Accordingly, a total shrinkage of 26.2% would be expected from the green body to the final sintering stage, and the obtained results aligned with this expectation. Additionally, no axial difference in shrinkage rate was observed, which indicates that the shrinkage was isotropic. With regard to the manufacturing method, the average shrinkage values for all axes were observed to differ between the 3D-printed and milled groups of both the cubic and cuboidal specimens. CAD/CAM methods are known to involve homogeneous particle distributions that result in uniform plastic shrinkages of approximately 25–30% on average. This is because pre-sintered zirconia particles are compressed in an infinite multiaxial direction before being cut and sintered [17].

The shrinkage rates of the 3D-printed cubic specimens were 19.88%, 21.99%, and 20.02% along the X-axis, Y-axis, and Z-axis, respectively. Unlike in the milling method, the shrinkage rate was significantly higher along the y-axis. The cuboidal specimens also exhibited an anisotropic shrinkage pattern with a significantly higher shrinkage rate along the Y-axis (21.88%) than along the X-axis (20.26%) or the Z-axis (19.72%). According to these results, the Y-axis exhibited the highest gravity-related shrinkage during 3D printing and was not involved in the axial change. The result is more affected by the direction of gravity during 3D printing than the axis during sintering. Similar to most other studies, the findings of this study also revealed a higher shrinkage rate along the 3D printing direction of the specimens than along the length or width direction.

DLP-based printing is a layer-by-layer process in which each layer is shaped before the prototyping platform is moved along the printing direction to complete the fabrication. To control the shrinkage rate in layer-by-layer fabrication, the material’s microstructure and the printing parameters must be optimized. Due to the difficulties in controlling certain parameters, the dimensional accuracy in the printing direction is compromised, which is accompanied by spreading compaction/densification of the powder within the layers [35]. Li et al. explained that the resin distribution in the height direction is sparse due to the presence of interfaces between the layers during 3D printing [36]. Additionally, Crivello et al. argued that the shrinkage may vary due to differences in the cure depth along different directions during 3D printing [37]. In this study, the 25 μm thick printed layers were examined using scanning electron microscopy images, which showed no cracks or voids in the cross-section. Although the quality of 3D-printed zirconia is high, the presence of interfaces between the layers implies that the distribution of resin between the layers during printing differs from that within the layers; this results in a non-ideal porosity distribution during the sintering process, which causes anisotropic shrinkage.

Unlike most studies, which have reported only linear shrinkage rates [38,39,40], this study presents shrinkage rates for specimens subjected to axis changes, as well as shrinkage rates at different sintering stages and along different axes. The findings demonstrate anisotropic shrinkage, with greater shrinkage occurring in the building direction during printing. Since shrinkage inevitably occurs during the sintering of the zirconia green body to the final product, additive manufacturing techniques for zirconia should be designed considering the associated shrinkage characteristics. The expansion rate for each axis should be varied depending on the direction. In this study, the standard deviation of the average shrinkage rate was the highest along the Y-axis, which suggests that the product may not be suitable for clinical applications, even if the shrinkage rate is compensated for during manufacturing. Additionally, the difference in refractive index between the resin and the zirconia in the zirconia suspension can cause light scattering and geometric overgrowth of products during additive manufacturing [41]. For complex-shaped products, pore release may be difficult, and the actual shrinkage rate may differ from the predicted rate [14]. To enable the use of 3D-printed zirconia products in clinical applications, additional research must focus on either accurate specimen enlargement considering the anisotropy of axial shrinkage rates or achieving isotropic contraction instead.

## 5. Conclusions

The shrinkage rate of the 3D-printed zirconia during sintering exhibited anisotropic behavior. Zirconia specimens fabricated via 3D printing showed a lower shrinkage rate and higher deviation than those manufactured through milling. The pre-sintering temperature or axis changes during sintering did not affect the average shrinkage rate. For the 3D-printed zirconia, the shrinkage rate was significantly higher along the building direction during printing than along the gravity direction during sintering.

Most studies have reported that 3D printing techniques involve different shrinkage rates along different axes. The shrinkage rate is known to be highest along the Z-axis, which is affected most by gravity. Therefore, in this study, the Z-axis of some of the specimens was switched to the X-axis during sintering to achieve isotropic shrinkage. However, this change did not affect the outcome significantly. Moreover, the Y-axis, which represents the direction of gravity during 3D printing, exhibited the highest shrinkage rate. These findings suggest that the shrinkage rate is affected more by the printing conditions than the sintering conditions.

## Figures and Tables

**Table 1:** Experimental groups for shrinkage rate evaluation.

Groups	Fabrication Method	Specimen Shape	Pre-Sinter Temperature (°C)	Axis Change	Count
MC	Milling	Cube		No	8
MR		Cuboid		No	8
C3	3D printing	Cube	1300	No	8
C3X				Yes	8
C4			1400	No	8
C4X				Yes	8
R3		Cuboid	1300	No	8
R3X				Yes	8
R4			1400	No	8
R4X				Yes	8

**Table 2:** Composition of zirconia slurry (vol%).

Zirconia	Acrylate			Photoinitiator	Dispersant	Total
	IBA	HDDA	PNPGDA	(Irgacure819)	(BYK-180)	
49.00	36.84			0.15	14.01	100

**Table 3:** Specifications of 3D printer.

Parameter	Value
Light direction	Top down
Energy source	385–400 nm
Layer thickness	50 μm
Exposure time per layer	10 s
Light intensity	110 mW/cm^2^
Z-axis lift distance	0.05 mm
Z-axis lift speed	0.4 mm/m
Blade distance	160 mm
Blade speed	10 mm/m

**Table 4:** Sintering schedule.

	Temperature (°C)	Rate (°C/min)	Holding Time (h:min)
Debinding	230	0.5	1:00
	380	0.5	2:00
	600	0.5	1:00
Pre-sintering	1040	1.0	1:00
	1300/1400	5.0	1:00
Final sintering	25	10.0	0:00
	600	10.0	0:30
	1000	10.0	1:00
	1300/1400	10.0	0:30
	1500	5.0	2:00

## Data Availability

The original contributions of the study are included in the article; further inquiries can be directed to the corresponding authors.

## Abbreviations

The following abbreviations are used in this manuscript:

CAD/CAM	computer-aided design/computer-aided manufacturing
LSPS	laser scanning projection stereolithography
MPSL	mask projection stereolithography
TGA	thermogravimetric analysis
DLP	digital light processing
M	subtractive manufacturing process
C	cubic 3D-printed specimen
R	cuboidal 3D-printed specimen
STL	standard tessellation language
LCM	light-curing resin molding
